# DEET Metabolite and Hearing Loss in United States Adults

**DOI:** 10.3390/toxics13090801

**Published:** 2025-09-20

**Authors:** Rae T. Benedict, Gregory M. Zarus, Franco Scinicariello, Henry G. Abadin, Roberta Attanasio

**Affiliations:** 1 Office of Innovation and Analytics, Agency for Toxic Substances and Disease Registry (ATSDR), Atlanta, GA 30341, USA; rbenedict@cdc.gov (R.T.B.); fes6@cdc.gov (F.S.); henryabadin@yahoo.com (H.G.A.); 2 Department of Biology, Georgia State University, Atlanta, GA 30303, USA; rattanasio@gsu.edu

**Keywords:** adult, deet, hearing loss, nutrition surveys, ototoxicity

## Abstract

Hearing loss (HL) causes numerous challenges for individuals. N,N-diethyl-meta-toluamide (DEET) is a common ingredient in insect repellants and some sunscreens. M-(diethylcarbamoyl) benzoic acid (DCBA) is a DEET metabolite measured in the urine of National Health and Nutrition Examination Survey participants. This study examines potential associations between HL and urinary DCBA in U.S. adults. Speech-frequency hearing loss (SFHL) was defined as an average hearing threshold above 25 dB across four frequencies (0.5, 1, 2, and 4 kHz). In comparison, high-frequency hearing loss (HFHL) was based on the average threshold above 25 dB at three higher frequencies (3, 4, and 6 kHz) in the better hearing ear. Hearing loss severity was categorized as normal (≤25 dB), mild (26–40 dB), and moderate or worse (≥41 dB). Higher DCBA exposure was significantly associated with increased odds of high-frequency hearing loss (HFHL). Participants in the highest exposure quartile had greater odds of HFHL (aOR = 2.34; 95% CI: 1.14–4.81), with a dose–response trend (*p* < 0.05) confirmed in sensitivity analyses controlling for chronic conditions and inflammation. Multinomial models further showed elevated odds of both slight/mild (aOR = 2.15; 95% CI: 1.05–4.42) and moderate/worse HFHL (aOR = 2.84; 95% CI: 1.10–7.37), supporting the robustness of the association. To our knowledge, this is the first report of HL being associated with a DEET metabolite in a nationally representative cross-sectional sample.

## 1. Introduction

Hearing loss is a widespread public health concern, affecting communication, cognition, employment, social interactions, and mental well-being [1]. In 2019, approximately 1.57 billion people—one in five worldwide—experienced hearing loss, with projections estimating this number will rise to 2.45 billion by 2050 [2].

While aging is a major factor, other contributors include noise exposure (occupational and recreational), infections, genetics, ototoxic medications, and chemical exposures [2]. Aromatic solvents, halogenated hydrocarbons, and metals, especially in workplace environments, have been linked to hearing impairment. Additionally, pyrethroid insecticides have been associated with hearing loss in both adolescents and adults [3,4].

DEET (N,N-diethyl-meta-toluamide) is a widely used insect repellent, effective in preventing mosquito bites. While generally considered safe, high-dose exposure has been associated with neurological, respiratory, cardiovascular, gastrointestinal, dermal, and ocular effects [5,6].

DEET enters the body primarily through dermal absorption, though inhalation via aerosol sprays is another exposure route. DEET is available in sprays, mists, lotions, and wipes, some of which include sunscreen, increasing the likelihood of frequent reapplication. After absorption, DEET is metabolized in the liver by cytochrome P-450 enzymes. Studies have detected DEET and its metabolites in several organs, including the brain, liver, kidneys, lungs, spleen, fat tissue, tears, and nasal cavity [7]. Its primary elimination pathway is urinary excretion, with DCBA and m-(ethylaminocarbonyl)benzoic acid (EACB) as key metabolites [5].

Despite concerns about DEET exposure, no studies have directly investigated its relationship with hearing loss. Using DEET as a biomarker of exposure may result in exposure misclassification and, therefore, unreliable results. In contrast, urinary metabolites like DCBA offer a more accurate and more sensitive biomarker of exposure than DEET itself [8]. To address this gap, we analyzed urinary DCBA levels and hearing loss in adults aged 20–69 years who participated in the 2015–2016 National Health and Nutrition Examination Survey (NHANES), which included audiometric evaluations. The aim of this study was to identify and assess substances that might contribute to ototoxic effects observed in the general population. A recent work suggested that exposure to relatively low environmental levels of benzene was associated with hearing loss in children [9].

## 2. Methods

Study Population

NHANES is a cross-sectional, nationally representative survey of the non-institutionalized civilian population of the US [10]. Counties, blocks, households, and individuals are systematically selected, and trained professionals conduct home interviews before inviting participants to undergo physical examinations—including blood and urine collection—at mobile examination centers. The NCHS Ethics Review Board approved all study procedures (Continuation Protocol #2011-17 Ethics Review Board Approval | National Health and Nutrition Examination Survey | CDC, accessed on 13 September 2025), and all participants provided written informed consent. For our analysis, we focused on adult participants aged 20 years and older from the 2015–2016 cycle who had complete audiometric measurements and urinary levels of DCBA, the primary metabolite of DEET. After excluding individuals with missing values for any covariates included in our multivariable models, we obtained a final analytic sample of 1078 participants.

### 2.1. Outcome Measure

Audiometric Measurements and Definition of Hearing Loss

Audiometric testing was performed in specially equipped, sound-treated rooms within the mobile examination centers by examiners trained by the National Institute for Occupational Safety and Health, following the standardized protocol established by the NCHS. Air conduction thresholds were measured for each ear at frequencies of 0.5, 1, 2, 3, 4, 6, and 8 kHz across an intensity range of −10 to 120 dB hearing level using calibrated audiometers. To assess reliability, a test–retest threshold was obtained at 1 kHz in each ear; any pair of measurements differing by 10 dB or more was considered invalid and excluded. Further details of the measurement techniques are available on the NCHS website (NHANES 2015–2016 Audiometry Procedures Manual; accessed 13 September 2025).

We excluded participants with an otoscopic screening exam of the ear canals and eardrum for excessive or impacted cerumen (ear wax), physical abnormalities, or collapsing external ear canals, ear compliance ≤ 0.2 mL, or pressure lower than −150 dekapascals (daPa). Speech-frequency hearing loss (SFHL) and high-frequency hearing loss (HFHL) were defined as pure tone audiometric (PTA) average of four frequencies (0.5, 1, 2, and 4 kHz) for SFHL and three frequencies (3, 4, and 6 kHz) for HFHL exceeding 25 dB in the better ear. HL degrees were categorized as normal ≤25 dB, mild = 26–40 dB, and moderate or worse ≥41 dB [11].

### 2.2. DEET Acid Biomarker

Urinary DCBA was measured by inductively coupled plasma-mass spectrometry using a multi-element analytical technique at the CDC, National Center for Environmental Health (NCEH), Division of Laboratory Sciences (DLS). The lower limit of detection (LLOD) for DEET acid was 0.20 ng/mL. Urinary concentrations below the LLOD were assigned the LLOD divided by the square root of 2, as recommended by the NCHS. Urinary DEET acid was categorized as weighted quartiles based on the distribution of urinary levels among the study population.

### 2.3. Covariates

The a priori covariates selected for inclusion in the models are associated with hearing impairment. These covariates include age (categorized in weighted tertiles: 20–35 years; 36–51 years, and 52–69 years), sex, race/ethnicity, exposure to occupational noise, off-work noise exposure, firearm noise, education, poverty income ratio (PIR), obesity, cigarette smoking, serum cotinine, diabetes, hypertension, and high-sensitivity c-reactive protein (hs-CRP) [1,2].

We obtained information about age (years), sex, race/ethnicity, and education from the household interview. Race/ethnicity was categorized as non-Hispanic white, non-Hispanic black, Hispanic, non-Hispanic Asian, and other race, including multiracial. PIR is a measure of socioeconomic status and represents the calculated ratio of the total family income to the federal poverty threshold after accounting for inflation and family size for the year of the interview. If the income data were missing, the values were not computed. PIR was dichotomized as PIR ≥ 1.3 and PIR < 1.3 based on the Health and Human Services poverty guidelines for eligibility to federal programs.

The histories of exposure to occupational noise, off-work noise, and firearm noise were obtained from the audiometry questionnaire, and each variable was dichotomized as “yes” or “no”.

Body mass index (BMI) was calculated as weight divided by height squared (kg/m^2^). The adult population was categorized as either normal/underweight (BMI < 25 kg/m^2^), overweight (BMI ≥ 25 to less than 30 kg/m^2^), or obese (BMI ≥ 30 kg/m^2^).

Smoking status was defined as never smoker (smoked <100 cigarettes ever), former smoker (not currently smoking, but has smoked ≥100 cigarettes ever), and current smoker (≥100 cigarettes in lifetime and currently smoking every day or some days). Serum cotinine and hs-CRP were obtained from their respective laboratory data modules (https://wwwn.cdc.gov/Nchs/Data/Nhanes/Public/2015/DataFiles/COT_I.htm [accessed 13 September 2025] and https://wwwn.cdc.gov/Nchs/Data/Nhanes/Public/2015/DataFiles/HSCRP_I.htm [accessed 13 September 2025], respectively) and were normalized by natural logarithm transformations. Participants were classified as having diabetes based on (1) the “yes” answer to the questions: “Other than during pregnancy, have you ever been told by a doctor or health professional that you have diabetes or sugar diabetes?” or “now taking insulin” or “now taking diabetic pills”; or (2) their hemoglobin A1c was ≥6.5%; or (3) their fasting (8–24 h) plasma glucose was ≥126 mg/dL. Participants were classified as having hypertension if their systolic blood pressure was ≥140 mmHg or diastolic blood pressure was ≥90 mmHg, or they were currently taking medication to lower high blood pressure.

### 2.4. Statistical Methods

Sas-callable Sudaan software (version 11; Research Triangle Institute; Research Triangle Park, Durham, NC, USA) was used to perform statistical analysis. A complex survey design was considered using chemical-specific subsample survey weights to obtain nationally representative estimates [12]. We used multivariate logistic regression to calculate adjusted odds ratios for having hearing loss (≥26 dB). Further, we also conducted analyses using multinomial logistic regression. This approach is used when the outcome variable has more than two unordered categories. For this analysis, we categorized hearing status into three distinct severity levels based on hearing thresholds: “normal hearing” (≤25 dB), “ slight/mild hearing loss” (≥26–40 dB), and “moderate/worse hearing loss” (≥41 dB). The multinomial regression allows for independently modeling and estimating adjusted odds ratios (ORs) for each degree of hearing loss (as distinct outcomes, compared with normal hearing ≤25 dB) in association with categorical DCBA exposures, as well as log-transformed DCBA. To account for variation in dilution in spot urinary samples, urinary creatinine was entered in the analyses as an independent variable, as suggested by previous studies [13].

We ran three models: Model 1 was adjusted for urinary creatinine (log transformed), age (tertiles) sex, and race/ethnicities; Model 2 was further adjusted for education, smoking status, serum cotinine (log transformed), PIR, obesity, exposure to occupational noise, off-work noise exposure and firearm noise; and as sensitivity analyses, the model was further adjusted with diabetes, hypertension and c-reactive protein (log transformed) (Model 3).

## 3. Results

Table 1 presents the weighted characteristics of the study population. The geometric mean (GM) age of the participants was 41 years, with 52.1% female. Non-Hispanic whites accounted for 64.0% of the total study group; 11.4% were non-Hispanic blacks, 15.4% were Hispanic, 5.8% were non-Hispanic Asian, and 3.5% belonged to “other race/ethnicity or designated themselves as multiracial”. Approximately 59% reported that they had never smoked, and 67.1% had attended college. Approximately 39.8% of the population was obese; 11.5% had diabetes; and 26% had hypertension. Exposure to occupational noise, off-work noise, and firearm noise was found in 29.5%, 17.7%, and 49.3% of participants, respectively.

The geometric mean (SE) urinary concentrations DCBA was 3.27 (0.54) ng/mL The weighted prevalence of speech-frequency hearing loss was 11.6% and that of slight/mild and moderate/worse SFHL grades were 7.7% and 3.8%, respectively. The weighted prevalence of high-frequency hearing loss was 30.9% and that of slight/mild and moderate/worse HFHL was 18.2% and 12.6%, respectively (Table 1).

No significant association was found between DCBA exposure and overall SFHL in the basic multivariate logistic regression analysis (Table 2).

However, multinomial logistic regression revealed that higher DCBA levels were significantly associated with increased odds of moderate/worse SFHL (Table 3). Specifically, when DCBA was modeled as a continuous (natural log-transformed) variable, the adjusted odds ratio (aOR) was 1.33 (95% CI: 1.01–1.76). Quartile analysis showed that participants in the highest DCBA quartile had substantially elevated odds of moderate/worse SFHL compared to those in the lowest quartile (aOR = 14.53; 95% CI: 3.71–5.96.96; Table 3, Model 2). No significant association was observed between DCBA and slight/mild SFHL. Sensitivity analyses adjusting for potential confounders supported the robustness of the association between DCBA and moderate/worse SFHL (Table 3, Model 3). Complementary analyses using natural log DCBA provided similar findings.

Multivariate logistic regression analyses indicated that individuals in the fourth quartile of DCBA had significantly greater odds of HFHL compared to the reference group (aOR = 2.34; 95% CI: 1.14–4.81; Table 4, Model 2), with evidence of a dose–response effect confirmed by a significant *p*-value for trend (*p* < 0.05). When DCBA was analyzed as a continuous natural log-transformed variable, a positive association was identified (aOR = 1.15; 95% CI: 1.01–1.32; Table 2, Model 2). Sensitivity analyses adjusting for chronic conditions (e.g., diabetes, hypertension) and inflammatory biomarkers confirmed the independent association between DCBA and HFHL (Table 4, Model 3).

Multinomial logistic regression further demonstrated that participants in the highest DCBA quartile had significantly increased odds of both slight/mild HFHL (aOR = 2.15; 95% CI: 1.05–4.42) and moderate/worse HFHL (aOR = 2.84; 95% CI: 1.10–7.37; Table 5, Model 2). When DCBA was modeled continuously, a significant linear association was detected only for moderate/worse HFHL. Although the association with slight/mild HFHL was directionally positive, it did not reach statistical significance. Sensitivity analyses affirmed the consistency and strength of the observed associations (Table 5, Model 3).

## 4. Discussion

To our knowledge, this is the first report of HL being associated with a DEET metabolite in a nationally representative cross-sectional sample. In this study, we observed a statistically significant positive association between DCBA metabolite concentration and high-frequency hearing loss (HFHL). Further analysis by severity of hearing loss revealed that DCBA was linked to an increased likelihood of both slight/mild and moderate/severe HFHL. While speech-frequency hearing loss (SFHL) showed a higher aOR with elevated DCBA levels, the association was not statistically significant. In contrast, a statistically significant positive association was observed between elevated DCBA and moderate to severe speech-frequency hearing loss. However, given the low prevalence of cases, these findings should be interpreted with caution. There are several reports of an association of insecticide and pesticide exposure with ototoxicity and hearing impairment [14,15]. Although pyrethroids are typically applied topically to clothing, tents, and mosquito nets for mosquito protection—resulting in minimal dermal absorption—studies have reported significant positive correlations between pyrethroid exposure and hearing loss [3,4].

Hearing relies on the conversion of sound waves into electrical signals via mechanoreceptors in cochlear inner hair cells, which depend on ionic gradients in the endolymph—an extracellular fluid rich in K+ (potassium) and low in Na+ (sodium). This ionic balance allows the passive flow of K+ into cells, contributing to sound perception. The basal cochlear hair cells detect higher frequencies, whereas the apical end responds to lower frequencies. Outer hair cells amplify the signal, which is then transmitted to the cochlear nucleus in the brainstem and the ascending auditory pathway [16,17].

One of the key biological effects of DEET is its role as a potassium and sodium channel blocker. In laboratory experiments using rat cortical neurons, researchers observed that DEET interferes with ion flow, which is essential for maintaining normal neuronal activity [18]. Given the importance of these channels in cochlear function and auditory transmission, this disruption raises concerns about its potential effects on hearing.

Additionally, studies suggest that DEET exposure alters blood–brain barrier (BBB) permeability, particularly in the brainstem—a critical region for auditory processing where sound signals are relayed from the cochlea to higher brain centers [19,20,21,22]. When the BBB is compromised, neurotoxic substances may infiltrate brain tissues and damage neurons responsible for sound transmission. Once DEET crosses the blood–brain barrier, it may disrupt neural signaling, compromise brainstem integrity, and cause neurodegenerative damage in both the brainstem and the Purkinje cell layer of the cerebellum, potentially affecting auditory function [21].

These findings highlight a broader neurotoxic potential, raising questions about how prolonged exposure might affect auditory pathways over time. Taken together, these biological mechanisms may suggest that DEET’s ability to disrupt ion channel activity, weaken the BBB, and contribute to neuronal degeneration could influence auditory function and hearing loss. While further investigation is necessary to fully understand the relationship, the existing evidence supports the need for closer scrutiny of DEET’s effects on neural and auditory health.

Increasingly frequent and intense extreme weather events such as heat waves, heavy rainfall, and droughts may influence the transmission patterns, geographic distribution, and re-emergence of vector-borne disease [23]. In this context, DEET remains a critical tool in disease prevention, serving as an effective insect repellent for personal protection.

However, data show that across the contiguous United States, the levels of UV rays increased between 2005 and 2015 [24]. Also, earlier data show that the total sunlight energy increased between 1991 and 2012 [24]. These changes suggest that people may have been exposed to more intense sunlight during these periods, thus prompting more frequent reapplication of sunscreen, including formulations containing DEET.

## 5. Limitations

While this study benefits from its large sample size and randomized selection, several limitations must be considered. The cross-sectional design prevents us from establishing a causal relationship between exposure and hearing loss, as it does not track changes over time. Hearing loss is a gradual process, and relying on a single biomarker measurement may not accurately reflect past exposure. Using urinary DEET metabolite concentrations as a marker for ototoxic exposure also introduces potential misclassification bias, despite evidence suggesting DEET can cross the blood–brain barrier and potentially affect auditory function. Another important limitation is that DEET has a short half-life, and exposure values are based on single spot urine analyses. A biomarker of longer-term exposure would have been preferred. Absorption of topically applied DEET is typically low (less than 10–20%) and has a short elimination half-life of 2.5 h [5,6]. However, the absorption rate can increase with higher concentrations, repeated use, or when combined with products like sunscreen, potentially increasing the risk of an unhealthy dose of DEET. In observational studies, confounding is a major source of bias that can obscure the true relationship between exposure and an outcome. Our analyses consistently found that the association between exposure and outcome remained stable across different adjustments. This consistency suggests that confounding is not a significant issue and strengthens the robustness of our findings. However, our analysis may not have accounted for all potential confounding factors, such as exposure to other environmental chemicals.

## 6. Conclusions

In our study, we found an association between hearing loss and a DEET metabolite in a sample of the U.S. population. Specifically, we observed a significant positive association between the concentration of the DCBA metabolite and high-frequency hearing loss (HFHL). This finding suggests that higher levels of DCBA increase the likelihood of both slight/mild and moderate/severe HFHL. Additionally, we identified a significant positive association between elevated levels of DCBA and moderate to severe speech-frequency hearing loss.

These findings in no way suggest that proper use of DEET is harmful as it is possible that the higher DCBA levels are from misapplications. Furthermore, there are scenarios where a person may unintentionally be exposed to DEET. For instance, a person may be unknowingly exposed when DEET is sprayed in a poorly ventilated enclosed space or when touching surfaces treated with DEET. However, DEET is an important repellent that helps prevent insect-borne diseases [25].

## 7. Recommendations

DEET provides many health benefits by reducing insect-borne diseases like West Nile virus, Lyme disease, Zika, and Rocky Mountain spotted fever. There are also alternative repellants to DEET [26].

Proper application involves the avoidance of application to open wounds and avoiding inadvertent ingestion. The addition of DEET to sunscreen might contribute to over-application of DEET. People who are unaware of DEET-containing skin products might apply those products (like sun block) in a manner different than if they knew it contained DEET. They might also apply DEET in addition to sunblock. One study suggested a greater DEET absorption when using sunscreen [27]. People need to be aware of the contents of skin products and follow proper application procedures. We recommend that people delay entering enclosed areas where DEET has been sprayed. Furthermore, upon returning indoors or when protection is no longer needed, it is recommended to wash your hands and any treated skin. Any clothing that has been treated with DEET should also be washed before wearing it again. Taking these precautions will help prevent unintended exposures (DEET (N,N-diethyl-meta-toluamide) | ToxFAQs™ | ATSDR; accessed 13 September 2025) [28]. Further study of the uptake, absorption, and elimination of DEET and its metabolites is recommended to help correlate the amount of DEET applied to urinary DEET or DCBA levels.

Should ototoxic effects from DEET or other exposures be suspected by health professionals, there are several management tools available for screening and mitigating additional hearing loss [14,29]. Periodic auditory screening efforts, in addition to exposure histories, could prevent hearing loss in more individuals.

## Figures and Tables

**Table 1:** Sample size and weighted characteristics of National Health and Nutrition Examination Survey (NHANES) 2015–2016 adult (20 to 69 years old) participants.

Characteristics	All Participants (*n* = 1078)
Age (years), GM (GSE)	41.2 (0.85)
Male, % (SE)	47.9 (1.8)
Female, % (SE)	52.1 (1.8)
BMI (kg/m^2^), GM (GSE)	28.47 (0.39)
Serum Cotinine (ng/mL), GM (GSE)	0.22 (0.05)
Poverty Income Ratio, GM (GSE)	2.53 (0.10)
Urinary Creatinine, (mg/dL), GM (GSE)	98.97 (3.84)
DCBA, (ng/mL), GM (GSE)	3.27 (0.54)
HS c-Reactive Protein (mg/L), GM (GSE)	1.53 (0.12)
Hearing Loss Categories	
HFHL ≤ 25 dB, % (SE)	69.1 (2.3)
HFHL ≥ 26 dB, % (SE)	30.9 (2.3)
HFHL ≥ 26–40 dB, % (SE)	18.2 (1.8)
HFHL ≥ 41 dB, % (SE)	12.6 (1.0)
SFHL ≤ 25 dB % (SE)	88.4 (1.3)
HFHL ≥ 26 dB, % (SE)	11.6 (1.3)
SFHL ≥ 26–40 dB, % (SE)	7.7 (2.0)
SFHL ≥ 41 dB, % (SE)	3.8 (1.0)
Poverty Level	
Poverty Income Ratio < 1.3, % (SE)	19.3 (1.7)
Poverty Income Ratio ≥ 1.3, % (SE)	80.7 (1.7)
Metabolic/Cardiovascular Status	
Diabetes, Yes, % (SE)	11.5 (1.6)
Hypertension, Yes, % (SE)	26.0 (2.4)
Noise Exposures	
Occupational, Yes, % (SE)	29.5 (1.9)
Off-work, Loud Noise, Yes, % (SE)	17.7 (1.3)
Firearms, Yes, % (SE)	49.3 (4.0)
Body Weight Status	
Underweight/Normal, % (SE)	31.8 (2.4)
Overweight, % (SE)	28.4 (1.4)
Obese, % (SE)	39.8 (2.7)
Smoking Status	
Current smoker, % (SE)	19.9 (1.3)
Former smoker, % (SE)	21.3 (2.1)
Never smoked, % (SE)	58.8 (2.0)
Education Level	
Less than high school, % (SE)	11.2 (1.6)
Completed high school, % (SE)	21.7 (1.7)
More than high school, % (SE)	67.1 (2.6)
Race/Ethnicity	
Non-Hispanic white, % (SE)	64.0 (4.1)
Non-Hispanic black, % (SE)	11.4 (2.2)
Hispanic, % (SE)	15.4 (3.1)
Non-Hispanic Asian, % (SE)	5.8 (1.5)
Other and Multi-race, % (SE)	3.5 (0.8)

Abbreviations: BMI, body mass index; DCBA, m-(diethylcarbamoyl) benzoic acid; HFHL, high-frequency hearing loss; SFHL, speech-frequency hearing loss. BMI = body mass index; DCBA = m-(diethylcarbamoyl) benzoic acid; HFHL = high-frequency hearing loss; SFHL = speech-frequency hearing loss.

**Table 2:** Multivariate logistic regression adjusted odds ratio, aOR (95% confidence interval) of having speech-frequency hearing loss (SFHL) and urinary DCBA levels in U.S. adults.

	Speech-Frequency Hearing Loss (≥26 dB)		
	Model 1	Model 2	Model 3
Cases/Controls (*n*)	129/939	129/939	129/938
DCBA ≤ 0.78 ng/mL	1.00 [Reference]	1.00 [Reference]	1.00 [Reference]
DCBA 0.79–2.55 ng/mL	1.48 (0.39, 5.58)	1.49 (0.46, 4.88)	1.52 (0.47, 4.87)
DCBA 2.56–10.31 ng/mL	1.94 (0.57, 6.54)	1.70 (0.53, 5.46)	1.77 (0.53, 5.90)
DCBA > 10.31 ng/mL	2.34 (0.81, 6.74)	2.44 (0.91, 6.52)	2.48 (0.91, 6.79)
*p*-Trend	0.38	0.28	0.29
DCBA (log)	1.12 (0.98, 1.30)	1.13 (0.96, 1.32)	1.13 (0.96, 1.33)
*p*-value	0.10	0.12	0.13

Notes: Model 1 adjusted for age (tertile), sex, race/ethnicity, and urinary creatinine; Model 2 adjusted as for Model 1 plus education, smoking status, blood cotinine, poverty income level, obesity, occupational noise, firearm noise, and off-work noise; Model 3 adjusted as for Model 2 plus diabetes, hypertension, and C-reactive protein. Statistically significant at *p* < 0.05. Abbreviations: dB, decibels; DCBA, m-(diethylcarbamoyl) benzoic acid; SFHL, speech-frequency hearing loss.

**Table 3:** Multinomial logistic regression adjusted odds ratio, aOR (95% confidence interval) of mild or moderate/worse speech-frequency hearing loss (SFHL), normal hearing, and urinary DCBA levels in U.S. adults.

	Mild SFHL (≥26–40 dB) vs. Normal Hearing (≤25 dB)			Moderate or Worse SFHL (≥41 dB) vs. Normal Hearing (≤25 dB)		
	Model 1	Model 2	Model 3	Model 1	Model 2	Model 3
Cases/Controls (*n*)	92/939	92/939	92/939	37/939	37/939	37/939
DCBA ≤ 0.78 ng/mL	1.00 [Reference]	1.00 [Reference]	1.00 [Reference]	1.00 [Reference]	1.00 [Reference]	1.00 [Reference]
DCBA 0.79–2.55 ng/mL	1.12 (0.29, 4.36)	1.21 (0.37, 3.94)	1.22 (0.38, 3.87)	3.55 (0.47, 26.65)	2.63 (0.40, 17.43)	2.94 (0.43, 19.95)
DCBA 2.56–10.31 ng/mL	1.13 (0.41, 3.14)	1.25 (0.38, 4.04)	1.27 (0.38, 4.23)	8.59 (1.02, 72.59) *	4.85 (0.90, 26.14)	5.21 (0.90, 30.12)
DCBA > 10.31 ng/mL	1.16 (0.32, 4.25)	1.14 (0.32, 4.11)	1.14 (0.31, 4.25)	13.85 (3.13, 61.28) *	14.53 (3.71, 56.96) *	16.49 (4.13, 65.85) *
DCBA (log)	1.05 (0.84, 1.32)	1.04 (0.83, 1.30)	1.04 (0.82, 1.31)	1.26 (1.03, 1.55) *	1.33 (1.01, 1.76) *	1.36 (1.02, 1.81) *

Notes: Model 1 adjusted for age (tertile), sex, race/ethnicity, and urinary creatinine; Model 2 adjusted as for Model 1 plus education, smoking status, blood cotinine, poverty income level, obesity, occupational noise, firearm noise, and off-work noise; Model 3 adjusted as for Model 2 plus diabetes, hypertension, and C-reactive protein. Statistically significant at *p* < 0.05. Abbreviations: * statistically significant, dB, decibels; DCBA, m-(diethylcarbamoyl) benzoic acid; SFHL, speech-frequency hearing loss.

**Table 4:** Multivariate logistic regression adjusted odds ratio, aOR (95% confidence interval) of having high-frequency hearing loss (HFHL) and urinary DCBA levels in U.S. adults.

	High-Frequency Hearing Loss (≥26 dB)		
	Model 1	Model 2	Model 3
Cases/Controls (*n*)	321/747	321/747	321/747
DCBA ≤ 0.78 ng/mL	1.00 [Reference]	1.00 [Reference]	1.00 [Reference]
DCBA 0.79–2.55 ng/mL	1.01 (0.48, 2.15)	1.03 (0.50, 2.10)	1.00 (0.49, 2.03)
DCBA 2.56–10.31 ng/mL	1.68 (1.00, 2.81) *	1.77 (1.05, 3.01) *	1.75 (1.04, 2.97) *
DCBA > 10.31 ng/mL	2.20 (1.13, 4.27) *	2.34 (1.14, 4.81) *	2.30 (1.14, 4.64) *
*p*-Trend	0.05	0.05	0.05
DCBA (log)	1.15 (1.02, 1.30) *	1.15 (1.01, 1.32) *	1.16 (1.01, 1.32) *
*p*-Value	0.03	0.03	0.04

Notes: Model 1 adjusted for age (tertile), sex, race/ethnicities, and urinary creatinine. Model 2 adjusted as for model 1 plus education, smoking status, blood cotinine, poverty level, obesity, exposure to occupational noise, firearm noise, and off-work noise. Model 3 adjusted as for Model 2 plus diabetes, hypertension, and C-reactive protein. Abbreviations: * statistically significant, *p* < 0.05. dB = decibels; DCBA = m-(diethylcarbamoyl) benzoic acid; HFHL = high-frequency hearing loss.

**Table 5:** Multinomial logistic regression adjusted odds ratio, aOR (95% confidence interval) of mild or moderate/worse high-frequency hearing loss (HFHL), normal hearing, and urinary DCBA levels in U.S. adults.

	Mild HFHL (≥26–40 dB) vs. Normal Hearing (≤25 dB)			Moderate or Worse HFHL (≥41 dB) vs. Normal Hearing (≤25 dB)		
	Model 1	Model 2	Model 3	Model 1	Model 2	Model 3
Cases/Controls (*n*)	180/747	180/747	180/747	141/747	141/747	141/747
DCBA ≤ 0.78 ng/mL	1.00 [Reference]	1.00 [Reference]	1.00 [Reference]	1.00 [Reference]	1.00 [Reference]	1.00 [Reference]
DCBA 0.79–2.55 ng/mL	1.05 (0.48, 2.30)	1.10 (0.55, 2.20)	1.05 (0.52, 2.09)	0.94 (0.29, 3.04)	0.89 (0.28, 2.85)	0.93 (0.30, 2.87)
DCBA 2.56–10.31 ng/mL	1.42 (0.85, 2.28)	1.53 (0.95, 2.46)	1.48 (0.93, 2.35)	2.25 (0.90, 5.58)	2.26 (0.95, 5.39)	2.31 (0.98, 5.44)
DCBA > 10.31 ng/mL	1.99 (1.03, 3.85) *	2.15 (1.05, 4.42) *	2.06 (1.01, 4.19) *	2.65 (1.08, 6.52) *	2.84 (1.10, 7.33) *	2.93 (1.13, 7.58) *
DCBA (log)	1.11 (0.97, 1.27)	1.12 (0.97, 1.29)	1.12 (0.97, 1.30)	1.21 (1.05, 1.39) *	1.23 (1.06, 1.43) *	1.23 (1.05, 1.43) *

Notes: Model 1 adjusted for age (tertile), sex, race/ethnicities, and urinary creatinine. Model 2 adjusted as for model 1 plus education, smoking status, blood cotinine, poverty income level, obesity, exposure to occupational noise, firearm noise, and off-work noise. Model 3 adjusted as for model 2 plus diabetes, hypertension, and C-reactive protein. Abbreviations: * Statistically significant, *p* < 0.05. dB = decibels; DCBA = m-(diethylcarbamoyl) benzoic acid; HFHL = high-frequency hearing loss.

## Data Availability

NHANES datasets may be obtained at https://wwwn.cdc.gov/Nchs/Nhanes/Search/default.aspx (accessed on 13 September 2025). Following this link, one may search for words and “quoted phrases” in selected fields (Data File Name, SAS Label, Variable Description, Variable Name) of Continuous NHANES (1999 and on) variables that have published documentation. Refer to the Survey Content Brochure (accessed on 13 September 2025) for the full content of Continuous NHANES. Auditory manuals are available at: (NHANES 2015-2016 Audiometry Procedures Manual; accessed 13 September 2025). Serum cotinine and hs-CRP were obtained from their respective laboratory data modules https://wwwn.cdc.gov/Nchs/Data/Nhanes/Public/2015/DataFiles/COT_I.htm [accessed 13 September 2025] and https://wwwn.cdc.gov/Nchs/Data/Nhanes/Public/2015/DataFiles/HSCRP_I.htm [accessed 13 September 2025], respectively.
